# Caregiving and allostatic load predict future illness and disability: A population-based study

**DOI:** 10.1016/j.bbih.2021.100295

**Published:** 2021-07-07

**Authors:** Stephen Gallagher, Bennett Kate M

**Affiliations:** a Department of Psychology, Centre for Social Issues Research, Study of Anxiety, Stress and Health Laboratory, University of Limerick, Limerick, Ireland; b Health Research Institute, University of Limerick, Castletroy, Limerick, Ireland; c School of Psychology, University of Liverpool, Eleanor Rathbone Building, Bedford Street South, Liverpool, UK

**Keywords:** Allostatic load, Caregiving, Disability, Health, Inflammation

## Abstract

**Background:**

Caring for sick or disabled relatives is a key model for understanding the effects of chronic stress on immunity/inflammation; biomarkers which are part of an index of allostatic load. Here, we examine whether caring and allostatic load are predictive of future illness/disability and if the association between caring and illness/disability is mediated by allostatic load.

**Method:**

Using data from the Understanding Society Wave 2 (2011) and Wave 9 (2017–2019) datasets in the UK, a sample of 471 of caregivers and 2,151 non-caregivers (all initially healthy) were compared on allostatic load and future illness/disability.

**Results:**

Caregivers had higher allostatic risk scores, for total as well as immune and non-immune biomarkers, and were more likely (23.3% vs 17.4%) to have an illness/disability in the future compared to non-caregivers. Moreover, caregiving was responsible for a 33% future illness/disability risk. Further, allostatic load was also predictive of excess risk (OR ​= ​1.18, 95% CI ​= ​1.08 – 1.26; *p* ​< ​.001); higher allostatic load was associated with increased risk of illness/disability in the future. In an unadjusted mediation model, allostatic load mediated the association between caregiving and future illness/disability. However, after controlling for confounding, the mediation became non-significant.

**Conclusions:**

These results confirm that caregiving and allostatic load are damaging for future health. Results are also discussed in relation to public health aspects of caregiving.

## Highlights

• Caregivers have a greater risk of future illness and disability relative to non-caregivers.
• Allostatic load is higher among caregivers.
• Risk of illness and disability continued beyond cessation of caring.
• Caregiving and allostatic load predicted future illness and disability.

## 1 Introduction

Providing care to a sick or disabled relative, caregiving, is a key paradigm for understanding the effects of chronic stress on immunity (Epel et al., 2018; Whittaker and Gallagher, 2019). Research has found caregiving negatively influences a variety of immune and endocrine parameters in both younger and older caregivers (Whittaker and Gallagher, 2019). Although, recent meta-analytic work suggests these effects are weak and of no clinical significance (Potier, Jean-Marie Degryse and de Saint-Hubert, 2017; Roth et al., 2019). As such more research and clarity on caregiving and health is needed.

A key criticism of previous caregiver and immunity studies are methodological critiques. For examples, there is often a lack of control for confounding factors such as caregivers health status, i.e. they may already have poorer health, as already be immunologically compromised, or have very small sample sizes which are prone to sampling selection biases (e.g. recruitment of caregivers who may be stressed and attending support groups, whereas those less stressed do not attend); they do not control for other health indices (e.g. medication) and behavioural lifestyle factors (e.g. diet, smoke, and exercise) that may indirectly influence outcomes (Davison et al., 2016; Denham et al., 2019; Fredman et al., 2015; Kohut, 2016; Smith et al., 2019).

In addition, to addressing these issues, our intention is to build on the previous research. In particular, we will explore the impact of caregiving on allostatic load and whether or not this is predictive of future illness and disability. To our knowledge this is the first study to examine this. Allostatic load is viewed as a measure of the cumulative burden on multiple physiological systems including the metabolic (e.g. blood pressure, high-density lipoprotein (HDL), endocrine (e.g. Dehydroepiandrosterone (DHEA), cortisol) and the immune (e.g. C-reactive protein) of the body as it attempts to adapt to life’s demands (McEwen and Stellar, 1993). Caregivers who are under higher strain have been found to be more vulnerable to allostatic load compared to those under less strain (Dich et al., 2015), and compared to non-caregivers controls their levels are statistically higher (Roepke et al., 2011). Allostatic load has been found to be predictive of illness and disability (Guidi et al., 2021), and as such, this may explain the increased health risk seen in informal caregivers (Pinquart and Sorensen, 2003; Vitaliano et al., 2003). This will be the focus on the present study.

In order to overcome issues of small sample size and selection bias, this study will use a longitudinal population-based study to examine the impact of caregiving on future risk of illness/disability and whether this is predicted by allostatic load. We expect that, after controlling for confounding, caregivers will have 1) higher scores of allostatic load, and 2) greater risk of future illness/disability. 3) The association between caregiving and illness/disability will be mediated by allostatic load.

## 2 Methods

### 2.1 Participants

Our data was obtained from Wave two (2011-13) and Wave 9 (2017–2019) of the Understanding Society study in the UK (Essex., 2010–12). The dataset is a stratified clustered random sample of households' representative of the UK general population. Biomedical measures and blood samples for Wave 2 were collected during a single nurse visit a few months after the survey data were collected, which took place in the participant's home 5 months after the wave 2 interview). The study has ethical approval and each participant gave informed consent.

**Fig. 1:**
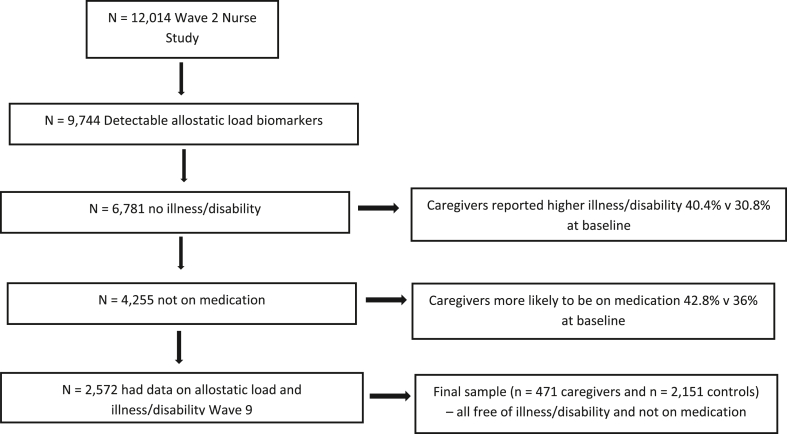
Participant selection flow diagram.

Information about caregiving was ascertained from two questions “Is there anyone living with you who is sick, disabled or elderly whom you care for or give special help to (for example, a sick, disabled or elderly relative/husband/wife/friend etc)?” and “Do you provide care for or service or help for any sick, disabled or elderly person not living with you?” Similar Yes/No format for both. People who answered ‘no’ to these caring questions served as our non-caregiver control group. We dichotomized several of our socio-demographic variables including relationship status (married/partnered vs single/divorced/widowed) education level (college education vs high school or less) and ethnicity (Caucasian/white vs other). Given the role of medication in influencing the immune and hormonal system (Rhen and Cidlowski, 2005) participants who reported taking medication except the contraceptive pill were excluded. Moreover, we also excluded those who had a long-standing illness or disability as baseline. See Fig. 1 for participant selection. To control for confounding through health behaviors we also considered variables that assessed smoking behaviour, alcohol intake, fruit and vegetable consumption, and exercise as these are known to influence immunity (Davison et al., 2016; Kohut, 2016). Participants were only included if they had detectable levels of biomarkers for assessment of allostatic load. Table 1 has socio-demographics and health characteristics for each group.

**Fig. 2:**
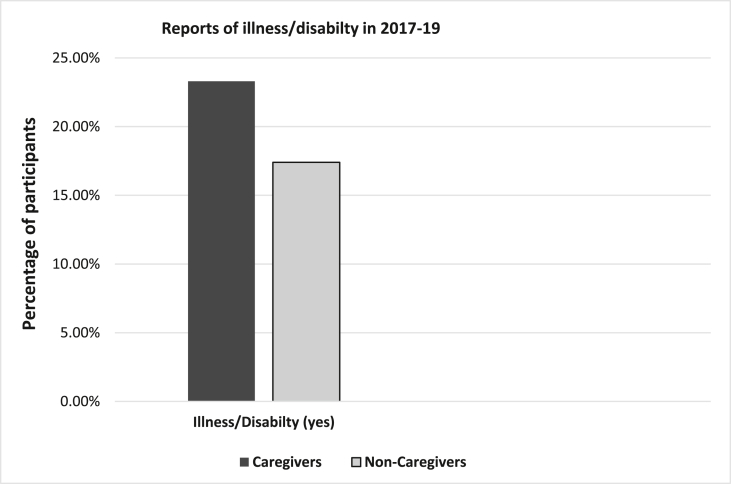
Percentage of Caregivers and Non-Caregivers who reported having an illness and disabilty.

**Table 1:** Sociodemographics, health behaviours and main outcome variables across caring groups.

Variable	Non-caregiver (*N* ​= ​2,151)	Caregiver (*N* ​= ​471)	Test of difference
Age	41.75 (13.78)	46.15 (13.65)	F (1, 2,306) ​= ​33.64, *p* ​< ​.001
Married/Partnered %	61.9	67.8	χ^2^ (1) ​= ​6.11, *p* ​= ​.01
Sex (female) %	44.7	52.9	χ^2^ (1) ​= ​11.06, *p* ​< ​.001
Ethnicity % (White)	91.1	91.2	χ^2^ (1) ​= ​0.43, *p* ​= ​.50
Education % (University level)	41.3	33.3	χ^2^ (1) ​= ​13.10, *p <*.001
Income (Monthly £)	1,982.04 (1,678.00)	1,982 (1,926.50)	F (1, 3,306) ​= ​0.00, *p* ​= ​.99
Alcohol (number days in last week)	2.92 (1.93)	2.94 (1.85)	F (1, 1,564) ​= ​0.16, *p ​= ​.68*
Walking 30min (days in last month)	10.95 (10.04)	11.74 (10.51)	F (1, 2,104) 0.67, *p ​= ​.41*
Fruit/Veg (servings per day)	3.30(1.55)	3.43 (1.60)	F (1, 2,576) ​= ​4.35, *p* ​= ​.037
Smoking %(yes)	40.6	38.6	χ^2^ (1) ​= ​0.50, *p* ​= ​.47
SF-12 Well-being	106.90(7.84)	105.61(1.55)	F (1, 2,493) ​= ​3.52, *p* ​= ​.04
Allostatic load	4.30 (1.61)	4.59 (1.66)	F (1, 2,358) ​= ​12.25, *p* ​< ​.001

### 2.2 Measures

#### 2.2.1 Illness/disability

Our main outcome measure, taken from Wave 9 dataset, and was captured by asking participants “Do you have any *long*-*standing* physical or mental impairment, *illness* or *disability*? By ‘*long*-*standing*’ I mean anything that has troubled you over a period of at least 12 months or that is likely to trouble you over a period of at least 12 months. Participants had to respond yes or no, coded as 1 and 0, respectively.

#### 2.2.2 Well-being

Caregivers also report poorer well-being relative to controls (Geng et al., 2018). Thus, to see future illness/disability varied by well-being we included this as a co-variate. The SF-12 is a 12-item survey for measuring functional health and well-being from patients’ point of view(Gandek et al., 1998). It consists of two subscales of mental and physical wellbeing. The scores on each scale range from 0 to 100, where a higher score represents better self-assessed health; here we summed the scores for a total score ranging from 0 to 200 as an index of well-being.

#### 2.2.3 Allostatic load

For the biological assessment (e.g. blood sampling, height, weight and blood pressure) a nurse visited each person’s home for blood draws and objective measurement. In accordance with the original definition (T. E. Seeman, McEwen, Rowe and Singer, 2001), we used 12 biomarkers representing four biological systems: the neuroendocrine system (DHEA-s); the immune system (insulin-like growth factor-1 (IGF1), C-reactive protein (CRP), and fibrinogen); the metabolic system (high-density lipoprotein (HDL), low-density lipoprotein (LDL), glycosylated haemoglobin (HbA1C), albumin, waist circumference and body mass index (BMI); and the cardiovascular system (systolic blood pressure (SBP), diastolic blood pressure (DBP). Biomarkers were then dichotomized into risk (high vs low) according to quartiles scores or sex specific risk (e.g. waist circumference) or established criteria (e.g. SBP/DBP 140/90 and BMI >25). For some (i.e., HDL cholesterol and DHEA-S) membership in the lowest quartile corresponded with the highest risk (T. E. Seeman et al., 2001). For IFG1 both high and low levels have been predictive of morbidity and mortality (Mikkel et al., 2009; Sanders et al., 2018) thus the top and bottom quartile were classified as high risk. These were dummy coded at 1 ​= ​high risk and 0 ​= ​low risk and summed together with higher scores indicating higher degree of risk (Brody et al., 2014; Hawkley et al., 2011; T. Seeman et al., 2004). The scores ranged between 1 and 10, with a mean of 4.35 (1.63). Allostatic load total was sub-divided into immune (e.g. CRP, IFF-1 and fibrinogen) and non-immune parameters (e.g. DHEAs, blood pressure, BMI etc) to examine which aspect had the greatest explanatory power.

### 2.3 Analytic Approach

Prior to statistical analyses, data were screened for assumptions of fit and normality and all *p*’s for Kolmogorov-Smirnov and Shapiro-tests were >. 05 and nor outliers were identified for allostatic load. Further, slight changes in degrees of freedom reflect missing data on some lifestyle or demographic data. Following this, test of differences were first conducted to examine group differences across caregiver groups. Following this, we found differences on age, gender and educational status which are predictive of allostatic load, thsu we created a propensity score matching variable by regressing these variables on our caregiver group. This new variable was then included as a co-variate in our main analyses. For we conducted hierarchal logistical regressions with covariates factors entered at Step 1, and caregivers and non-caregiver groups at Step 2, and well-being in Step 3 and allostatic load in Step 4. Partial Eta-squared (η^2^), R^2^ and odds ratio (OR) were used as an indicators of effect size. To see whether the association between caregiving and future illness/disability was mediated by allostatic load, we also tested a mediation model (using model 4 in Process)(Hayes, 2017).

## 3 Results

### 3.1 Preliminary analyses

There were no differences between caregivers inside the home, outside the home and dual caregivers on future illness/disability,χ2 (2) ​= ​1.01, *p* ​= ​.60. Thus, these groups were pooled and examined as one caregiving group. As can be seen from Table 1, non-caregivers were younger, and more likely to have a university degree. Caregivers were also more likely to be women, and married/partnered and to eat more fruit and vegetables per day. The groups also differed on allostatic load, with caregivers having a higher risk score, with a partial Eta-squared (η^2^), of 0.005. It is worth nothing that dual carers had a higher allostatic load compared to the other caring groups. Caregiver were also more likely to report poorer well-being (SF-12), (η^2^), of 0.002. Further, the differences between caregivers and non-caregivers were evident for both immune and non-immune measures of allostatic load (all p’s ​< ​0.001).

In terms of our outcome, a higher percentage of caregivers reported having a long-standing illness/disability in 2017–19 (See Fig. 2), χ2 (1) ​= ​9.54, *p* ​= ​.002, compared to non-caregivers*.* Moreover, we also explored whether this differed whether or not caregivers were still caring or not in 2017–19; these groups did not differ, *p* ​= ​.77.

### 3.2 Predictors of future illness/disability in caregivers

In Step 1 of our hierarchical logistics regressions we entered propensity score, age, sex, marital status and eating fruit and vegetables as these were likely confounding. This was followed by entering well-being at Step 2, caregiver group at Step 3, and allostatic load in Step 4. As seen in Table 2, age, marital status and education were significant predictors of future illness/disability, such that those who were younger and married/partnered and degree educated having a lower risk. While in Step 2, after controlling for these factors, well-being was also significant with those having better well-being having a lower risk of future illness/disability. In Step3, caregiving remained significant, with caregivers having a 32% greater risk of future illness/disability (OR ​= ​1.32, 95% CI ​= ​1.02 – 1.84, *p* ​= ​.03). In Step 4, allostatic load was also significant, contributing to 18% of this excess risk. In sensitivity analysis we entered immune and non-immune indices of allostatic load simultaneously in Step 4, and the non-immune parameters proved predictive of future illness/disability(OR ​= ​1.23, 95% CI ​= ​1.12 – 1.36, *p* ​= ​.03). The immune indices were non-significant.

**Table 2:** Summary of hierarchical logistic regressions for predicting illness/disability in 2017–2019 across caregivers and non-caregivers.

Variables	B	OR	*P*	95%CI	95%CI
Step 1					
Propensity Match	−5.58	.004	.29	0.00	138.00
Age	.04	1.04	**.033**	1.003	1.07
Sex	.17	1.18	.57	0.69	2.03
Married/Partnered	.32	1.38	**.025**	1.04	1.83
Education	.34	1.41	**.026**	1.04	1.91
Fruit/Vegetables	.01	1.01	.74	0.94	1.09
**Step 2**					
Well-being (SF-12)	-.04	0.96	**.001**	0.94	0.97
**Step 3**					
Caregiving groups	.33	1.37	.**036**	1.02	1.84
**Step 4**					
Allostatic Load	.16	1.18	**.001**	1.08	1.26

This analysis was followed by a mediational model (Model 4 in PROCESS) to see if the association between caregiving and illness/disability was mediated by allostatic load. In unadjusted analyses, we found evidence of mediation (indirect effect, B ​= ​0.08 [0.035, 0.133]) such that caregivers who had higher allostatic risk also had greater illness/disability in the future. However, after controlling for confounding the mediation became non-significant (B ​= ​0.02 [-0.0089, 0.0593]. We repeated the same for non-immune indices and this was also non-significant, (B ​= ​0.02 [-0.0077, 0.0598].

## 4 Discussion

The present study confirmed that caregivers had higher levels of allostatic load compared to non-caregiving controls. This was evident for both immune and non-immune indices of allostatic load. Further, as predicted, we found that a higher percentage of caregivers were more likely to report future illness/disability in 2017–2019 (22.5% vs 16.7%). After controlling for several potential confounding factors (e.g. age, sex, education, relationship status and fruit and vegetable intake) well-being, caregiving and allostatic load proved predictive. Following adjustment, being in the caregiving category was associated with a 33% increased risk of future illness/disability while allostatic contributed to an18% excess risk. In sensitivity analyses of immune and non-immune indices, it was the non-immune (e.g. DHEA-s, blood pressure, obesity, and cholesterol etc.) that proved predictive. Moreover, with over 6.5 million caregivers in the United Kingdom (CarersUK., 2021), this extra 5.8% caregiver v non-caregiver group difference in future illness/disability equates to approximately 377,000 family caregivers who are negatively impacted. Thus, our findings underscore the importance of why caregiving should be considered a public health concern (Shaji and Reddy, 2012).

The increased risk of illness and disability in caregivers is similar to that found elsewhere (Gallagher and Hannigan, 2014; Pinquart and Sorensen, 2003). Here, we found it was evident across caregivers in general and not just illness specific or spousal caring. Moreover, this was also irrespective of whether they were still caring at follow-up or caregivers who has stopped caring. Thus, the health impact of caring appears to extend beyond cessation of caring.

While for allostatic load our findings concur with smaller scale studies showing that caregivers have higher risk of allostatic load (Roepke et al., 2011). While in those studies it was for caregivers of Alzheimer’s here is was for caregivers in general. They are also comparable to studies demonstrating the negative effects of caregiving on immune (Gallagher et al., 2009; Lovell and Wetherell, 2011) and non-immune indices (Fredman et al., 2010; Gallagher and Hannigan, 2015). We also found that allostatic load, in particular non-immune indices, was predictive of future risk of illness/disability, and in our unadjusted models it was found to mediate the association between caregiver-status and illness/disability. Albeit, in our adjusted models it became non-significant, implying that there may be other, and likely interactive, pathways underlying this association (Whittaker and Gallagher, 2019). Moreover, while caregivers who cared inside the home, outside the home or who were doing both (dual carers) did not differ on future illness/disability, dual carers had a higher allostatic load, suggestive or greater physiological risk. This dual caring cohort is often neglected in caregiving and biomarkers studies and our findings suggest they may be worthy of further enquiry.

There are several limitations of the present study including the lack of care recipient illness type; caregiver stress has been found to vary across disability types (Gallagher et al., 2018). Also there may be other unmeasured factors important for caregiver health (e.g. caregiver personality, levels of social support, level of respite) that were not considered. While, these were not available in this dataset these factors could be explored further as studies have found these to be important for caregiver health (Whittaker and Gallagher, 2019). We did not examine the type of illness or disability reported and future research should examine whether there are particular types of conditions that caregivers are more prone to. Moreover, the idea behind allostatic load is that stress/burden accumulates over time and increases *wear and tear* on physiologic systems and our narrow window of assessment of caregiving, i.e., five months prior to blood assessment may not be an ideal measure of the chronicity of caregiving. Further, while our findings were significant, especially after controlling for confounding, the effect sizes are relatively small and as such our results should be interpreted with caution. There are also several strengths to the study including it is much larger than the vast majority of caregiver biomarkers studies, and it had an unbiased sample. It also controlled for a multitude of confounding factors from health, lifestyle and socio-demographic factors which appeared important.

In conclusion, the current study extends on the previous caregiving literature in several ways. There is an excess risk of future illness/disability in caregivers relative to non-caregivers. The risk was evident irrespective of cessation of caregiving, perhaps suggestive of a scarring effect. We also found that while caregivers has lower allostaic load this did not mediate the association between caregiving and future illness/disability. Moreover, we find these effects for did not vary by caring location (inside the home, outside the home or both) and withstood adjustment for several confounding factors.

## Declaration of competing interest

The authors have no conflicts of interest.
